# Prognostic Factors Impacting Surgical Resection Outcomes in Elderly Patients With Brain Metastasis

**DOI:** 10.1002/kjm2.70099

**Published:** 2025-08-26

**Authors:** Yu Chang, Heng‐Juei Hsu, Chia‐En Wong, Junmin Song, Kuo‐Chang Huang, Liang‐Chao Wang, Chih‐Hao Tien, Chih‐Yuan Huang, Po‐Hsuan Lee, Chi‐Chen Huang, Jung‐Shun Lee

**Affiliations:** ^1^ Section of Neurosurgery, Department of Surgery, National Cheng Kung University Hospital, College of Medicine National Cheng Kung University Tainan Taiwan; ^2^ Department of Neurosurgery Tainan Municipal Hospital (Managed by Show Chwan Medical Care Corporation) Tainan Taiwan; ^3^ Center of Transformative Bioelectronic Medicine, College of Medicine National Cheng Kung University Tainan Taiwan; ^4^ Department of Medicine Jacobi Medical Center, Albert Einstein College of Medicine Bronx New York USA; ^5^ Department of Neurosurgery Chiayi Christian Hospital Chiayi Taiwan; ^6^ Institute of Basic Medical Sciences, College of Medicine National Cheng Kung University Tainan Taiwan; ^7^ Department of Cell Biology and Anatomy, College of Medicine National Cheng Kung University Tainan Taiwan

**Keywords:** brain metastasis, elderly, surgical resection, survival

## Abstract

Brain metastases (BM) among elderly patients with cancer are increasing, and decision‐making for treatment is complicated by comorbidities. This study aimed to identify prognostic factors that can help make informed decisions regarding surgical resection in elderly patients with BM. We retrospectively included elderly patients (65 years or older) with newly diagnosed BM who underwent surgery. We conducted survival analyses and Cox regression analyses to identify potential independent predictors of poor survival. A total of 124 elderly patients with BM undergoing surgical resection were enrolled. In the multivariate analysis, male sex (HR: 1.96, 95% CI: 1.22–3.13), ECM (HR: 2.97, 95% CI: 1.82–4.85), BM in eloquent locations (HR: 1.64, 95% CI: 1.02–2.64), KPS deterioration (HR: 1.93, 95% CI: 1.20–3.10), and mFI‐5 equal to or greater than 2 (HR: 2.10, 95% CI: 1.12–3.95) were associated with poor overall survival. Conversely, receiving systemic treatment after the diagnosis of BM showed a significant overall survival benefit (HR: 0.45, 95% CI: 0.28–0.70). Elevated SII (HR: 1.99, 95% CI: 1.02–3.90) was significantly associated with poor survival, while elevated PNI (HR: 0.56, 95% CI: 0.33–0.94) indicated better survival. Clinicians should adopt a personalized approach when selecting treatment options for elderly patients with BM, considering BM location, the presence of ECM, comorbidities, and suitability for postoperative systemic treatment. Evaluating preoperative nutritional and inflammatory status and monitoring performance status pre‐ and postoperatively are needed, as these factors may affect prognosis.

## 1 Introduction

The global elderly population is increasing owing to improvements in healthcare [1, 2]. With aging, there is an exponential increase in cancer incidence, resulting in a surge in the number of elderly patients with cancer [3]. Advancements in cancer diagnostics and therapeutic techniques have improved the survival of these patients [4], leading to an increase in the incidence of brain metastasis (BM) from primary cancer. The development of multidisciplinary treatment modalities is crucial for the effective management [5, 6]. BM in the elderly population carries significant clinical implications, often leading to neurological deterioration and a decline in quality of life. Most elderly patients exhibit lower performance status, multiple comorbidities, restricted physiological reserves, and age‐related neurocognitive deficits that constrain and limit the options for aggressive interventions [7, 8].

Local treatments for BM, including surgical resection with adjuvant radiation therapy (RT) or RT alone [9]. Surgical resection provides immediate relief of the mass effect, direct tumor removal, and reduction of peritumoral edema [10]. We focused on surgical resection as it offers immediate benefits and addresses the limitations of other modalities like SRS stereotactic radiosurgery (SRS) and whole‐brain RT [11].

While many studies have investigated the prognostic factors for elderly patients undergoing surgical resection for primary brain tumors, decision‐making for elderly patients with BM is more complex because of their higher burden of comorbidities compared to those with primary brain tumors [6, 12]. In addition, biomarkers such as the neutrophil‐to‐lymphocyte ratio (NLR) [13], platelet‐to‐lymphocyte ratio (PLR) [14], prognostic nutritional index (PNI) [15], and systemic immune‐inflammation index (SII) [16], limited studies have yet to determine which group of elderly patients would yield better survival outcomes after surgical resection for BM. This study aimed to investigate the impact of patient and perioperative characteristics and prognostic indicators on survival outcomes of elderly patients undergoing surgical resection of BM.

## 2 Materials and Methods

We retrospectively included patients aged ≥ 65 years with newly diagnosed BM who underwent surgical resection for BM and postoperative whole‐brain RT at our institution between January 2010 and December 2023. The surgical goal for these patients was gross total tumor removal, as those who only underwent a biopsy were excluded from this study. For patients with multiple brain metastases, our surgical team focused on resection of the largest lesion, as it is often the most symptomatic and represents the greatest threat to the patient's neurological function. This study was approved by the institutional review board, and written informed consent was obtained from all participants. The work has been reported in line with the STROCSS criteria [17]. Patient data were collected from medical records, including patient characteristics, cancer status, survival time after surgery, presence of systemic treatments, synchronous or metachronous presentation of BM, presence of extracranial metastasis (ECM), number and size and location of BM, performance status, and the modified 5‐item frailty index (mFI‐5). Eloquent locations include the motor and sensory cortices, visual and speech centers, basal ganglia, thalamus, internal capsule, brainstem, and dentate nucleus. Maximum diameter of BM and edema region were measured using preoperative T2‐weighted FLAIR images. The presence of preoperative BM hemorrhage was determined using preoperative MRI and intraoperative findings.

Preoperative laboratory data was used to calculate the NLR, PLR, PNI, and SII with the calculation method is the same as in previous literature [13, 14, 15, 16]. These variables were treated as continuous measures in our analysis. We then utilized the receiver operating characteristic (ROC) curve to determine the optimal cutoff for each inflammatory and nutritional marker [18]. Additionally, we conducted a subgroup analysis for the most prevalent primary tumor site.

### 2.1 Statistical Analysis

R Studio (R 4.4.0) and its packages were used for data analysis. We utilized Kaplan–Meier survival curves and univariate Cox regression proportional hazard models to identify potential independent predictors of poor survival in geriatric patients undergoing surgical treatment for BM. For multivariate Cox regression proportional hazard analysis, we conducted variable selection by selecting models with low Akaike information criteria (AIC) with the “glmulti” package [19, 20]. For our analyses, the hazard ratio (HR) and 95% confidence interval (CI) using two‐sided *p*‐values were calculated, with statistical significance considered at a *p*‐value less than 0.05.

## 3 Results

### 3.1 Patient Characteristics

**FIGURE 1:**
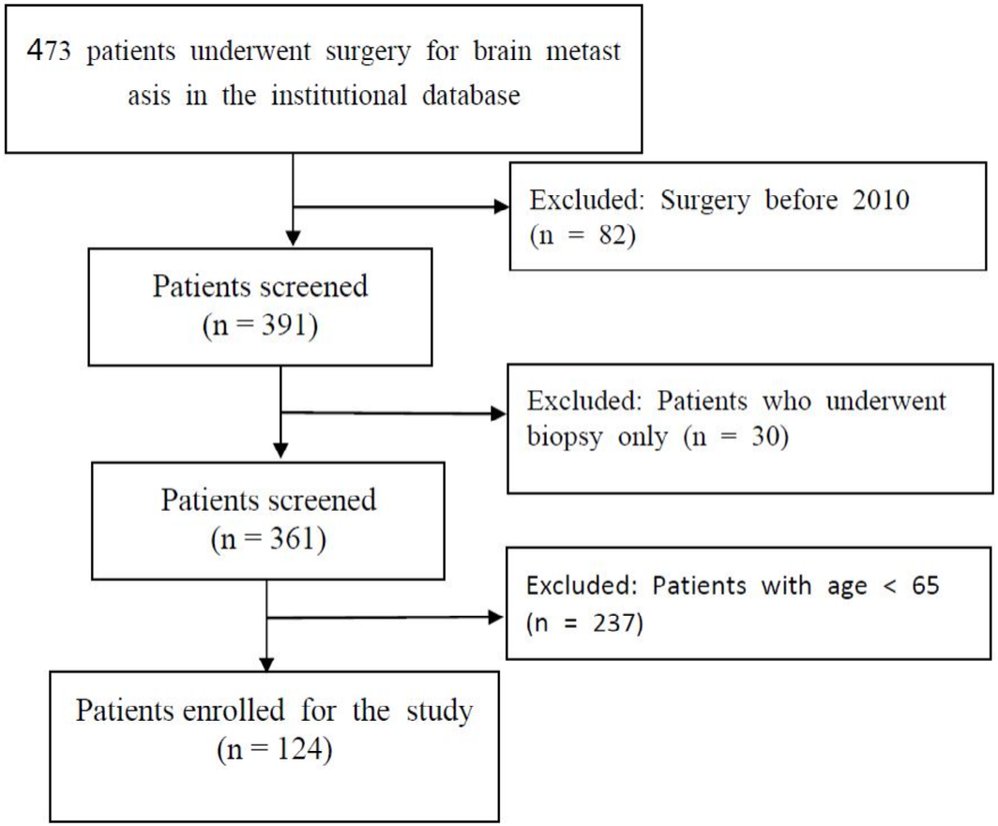
Diagram of the patient selection process.

A total of 124 elderly patients who underwent surgery for BM were included. The detailed patient selection process is depicted in Figure 1. Of these patients, 71 (57.2%) were male, with a mean age of 71.2 years. The most common primary cancer site was non‐small cell lung cancer (NSCLC) (72). At the time of craniotomy, 50 patients (40.3%) showed evidence of ECM. Thirty‐four patients (27.4%) had synchronous BM, and 20 patients (16.1%) had multiple BM (≥ 3 intracranial lesions). Of all the patients, 97 (78.2%) had supratentorial BM and 53 (42.7%) had BM involved in the eloquent area. The median overall survival was 9.48 months after surgery. Additional information is presented in Table 1.

**TABLE 1:** Demographic of elderly receiving surgery for BM.

Characteristic	*n* (%)	Characteristic	*n* (%) or mean (SD)	Characteristic	*n* (%) or mean (SD)
Primary cancer site		Patient characteristics		BM characteristics	
NSCLC	72 (58.1%)	Mean age at surgery	71.2	ECM	50 (40.3)
CRC	10 (8.1%)	Male	71 (57.2)	BM number: 1	88 (70.9)
Breast	12 (9.7%)	Median survival, (months)	9.48	BM number: 2	16 (12.9)
HCC	7 (5.6%)	Controlled primary tumor status	64 (51.6)	BM number ≥ 3	20 (16.1)
Esophageal	2 (1.9%)	Pre‐OP systemic tx	73 (58.8)	Max tumor diameter, cm	3.48 (1.28)
Gastric	1 (0.9%)	Post‐OP BM systemic tx	78 (62.9)	Max edema diameter, cm	6.17 (2.74)
Head and neck	3 (2.8%)	Functional dependent (RPA:3)	27 (21.7)	Eloquent area involvement	53 (42.7)
RCC	2 (1.9%)	Pre‐OP KPS	68.3 (9.14)	Supratentorial only	97 (78.2)
Gynecological	7 (5.6%)	Post OP KPS	61.8 (18.1)	Hemorrhage	34 (27.4)
Melanoma	2 (1.6%)	KPS deterioration	43 (34.7)	Functional dependent (RPA:3)	27 (21.7)
Bladder	2 (1.6%)	mFI‐5: 0	48 (38.7)		
Prostate	4 (3.8%)	mFI‐5: 1	42 (33.9)		
		mFI‐5 ≥ 2	34 (27.4)		
		WBC (10^3^/μL)	8.25 (4.0)		
		Platelet (10^3^/μL)	226.5 (86.9)		
		Neutrophil percentage	74.9 (12.4)		
		Lymphocyte percentage	16.1 (10.2)		
		Albumin (g/dL)	3.7 (0.6)		

Abbreviations: BM, brain metastasis; CRC, colorectal cancer; ECM, extracranial metastases; HCC, hepatocellular carcinoma; KPS, Karnofsky performance status; Mfi‐5, modified 5‐item frailty index; NSCLC, non‐small‐cell lung cancer; RPA, recursive partitioning analysis.

### 3.2 Cutoff for NLR, PLR, PNI, and SII

The ROC curve was plotted, and the optimal cutoff for each marker was calculated. The cutoff for NLR was 5.35 (sensitivity: 0.696, specificity: 0.545, AUC: 0.5799); for PLR, it was 301.5 (sensitivity: 0.435, specificity: 0.733, AUC: 0.557); for PNI, it was 38.5 (sensitivity: 0.957, specificity: 0.277, AUC: 0.589), and for SII, it was 880 (sensitivity: 0.784, specificity: 0.446, AUC: 0.581). These cutoffs were similar to those reported previously [21, 22, 23, 24].

### 3.3 Univariate Cox Regression & Kaplan–Meier Survival Analysis

**FIGURE 2:**
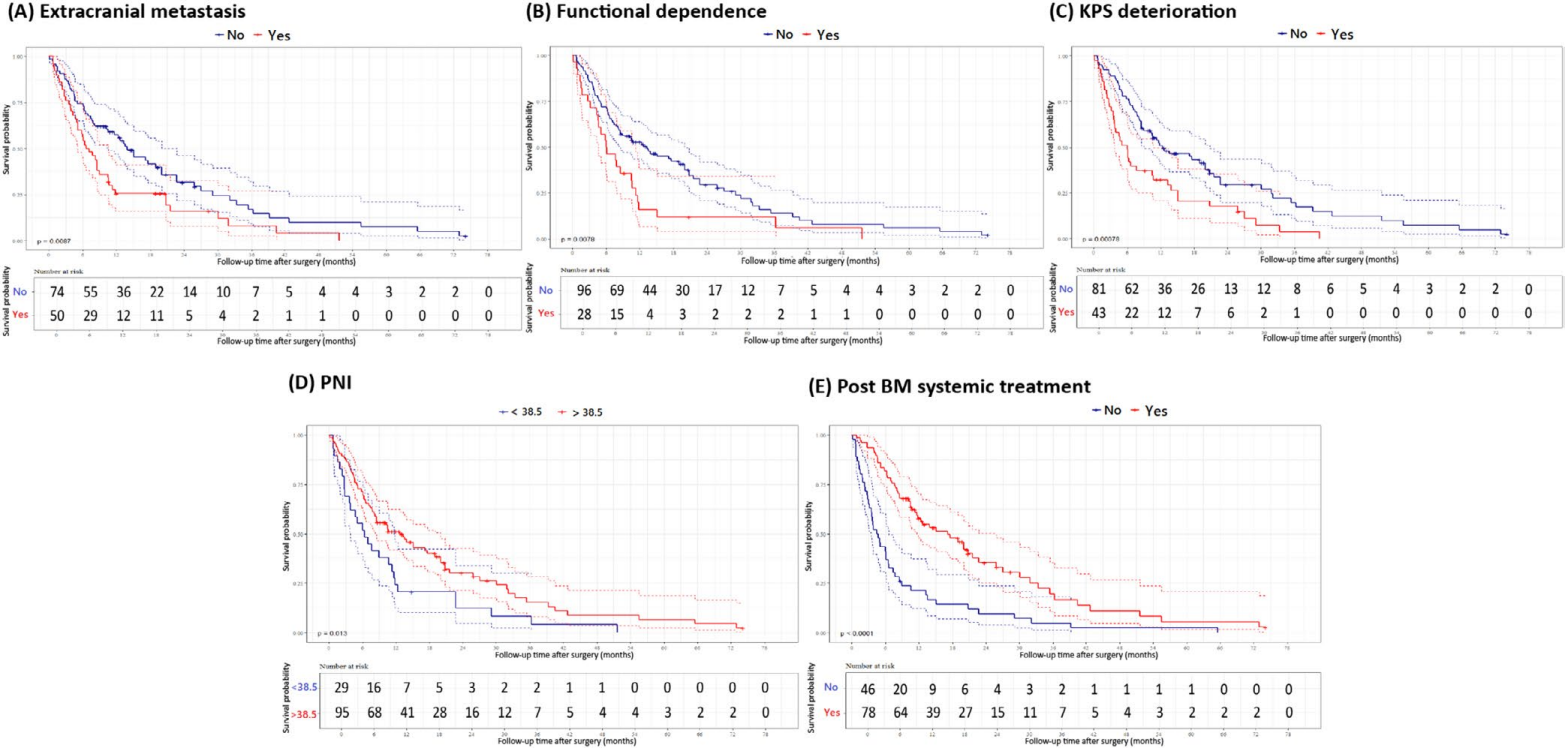
Kaplan–Meier survival curve of prognostic factors.

In univariate analysis, ECM (HR: 1.70, 95% CI: 1.14–2.55, Figure 2A), preoperative functional dependence status (RPA: 3) (HR: 1.83, 95% CI: 1.17–2.88, Figure 2B), and postoperative deterioration of KPS (HR: 2.00, 95% CI: 1.33–3.02, Figure 2C) were associated with poor survival. Elevated PNI was significantly associated with better survival (HR: 0.58, 95% CI: 0.37–0.900, Figure 2D) as well as receiving postoperative systemic treatment (HR: 0.38, 95% CI: 0.25–0.56, Figure 2E). Further details are provided in Table 2.

**TABLE 2:** Cox regression of prognostic factors.

Characteristics	Univariate Cox regression			Multivariate Cox regression		
	HR	95% CI	*p*	HR	95% CI	*p*
Age	1.00	0.97–1.04	0.855	0.96	0.92–1.01	0.111
Male	1.50	1.00–2.25	0.049*	1.96	1.22–3.13	0.005*
RPA: 3	1.83	1.17–2.88	0.009*			
Controlled primary tumor status	1.11	0.74–1.65	0.617			
ECM	1.70	1.14–2.55	0.009*	2.97	1.82–4.85	< 0.001*
Eloquent area involvement	1.34	0.91–1.99	0.142	1.64	1.02–2.64	0.041*
KPS deterioration	2.00	1.33–3.02	< 0.001*	1.93	1.20–3.10	0.007*
BM bleeding	1.18	0.76–1.83	0.463			
Number of BM						
1	Ref					
2	1.08	0.62–1.90	0.785	0.61	0.31–1.17	0.135
≥ 3	0.75	0.43–1.32	0.318	0.59	0.32–1.09	0.09
Synchronous BM	0.93	0.60–1.43	0.731			
Infratentorial involvement	1.33	0.82–2.16	0.243			
Tumor size (cm)	1.00	0.98–1.01	0.61			
Edema size (cm)	1.00	1.00–1.01	0.976			
Pre BM systemic tx	0.94	0.63–1.40	0.751			
Post BM systemic tx	0.38	0.25–0.56	< 0.001*	0.45	0.28–0.70	< 0.001*
Functional dependence	2.03	1.31–3.15	0.002*			
mFI‐5						
0	Ref					
1	1.60	1.00–2.56	0.052	1.58	0.94–2.67	0.084
≥ 2	1.53	0.94–2.50	0.087	2.1	1.12–3.95	0.021*
SII ≥ 880	1.18	0.80–1.76	0.405	1.99	1.02–3.90	0.044*
PNI ≥ 38.5	0.58	0.37–0.90	0.015*	0.56	0.33–0.94	0.027*
PLR ≥ 301.5	1.29	0.82–2.03	0.265			
NLR ≥ 5.35	0.98	0.66–1.45	0.908	0.56	0.29–1.07	0.079

Abbreviations: BM, brain metastasis; CI, confidence interval; ECM, extracranial; HR, hazard ratio; KPS, Karnofsky performance status; mFI‐5, modified 5‐item frailty index; NLR, neutrophil‐to‐lymphocyte ratio; PLR, platelet‐to‐lymphocyte; PNI, prognostic nutrition index; RPA, recursive partitioning analysis; SII, systemic inflammation index.

* Statistically significant.

### 3.4 Multivariate Cox Regression Analysis

**FIGURE 3:**
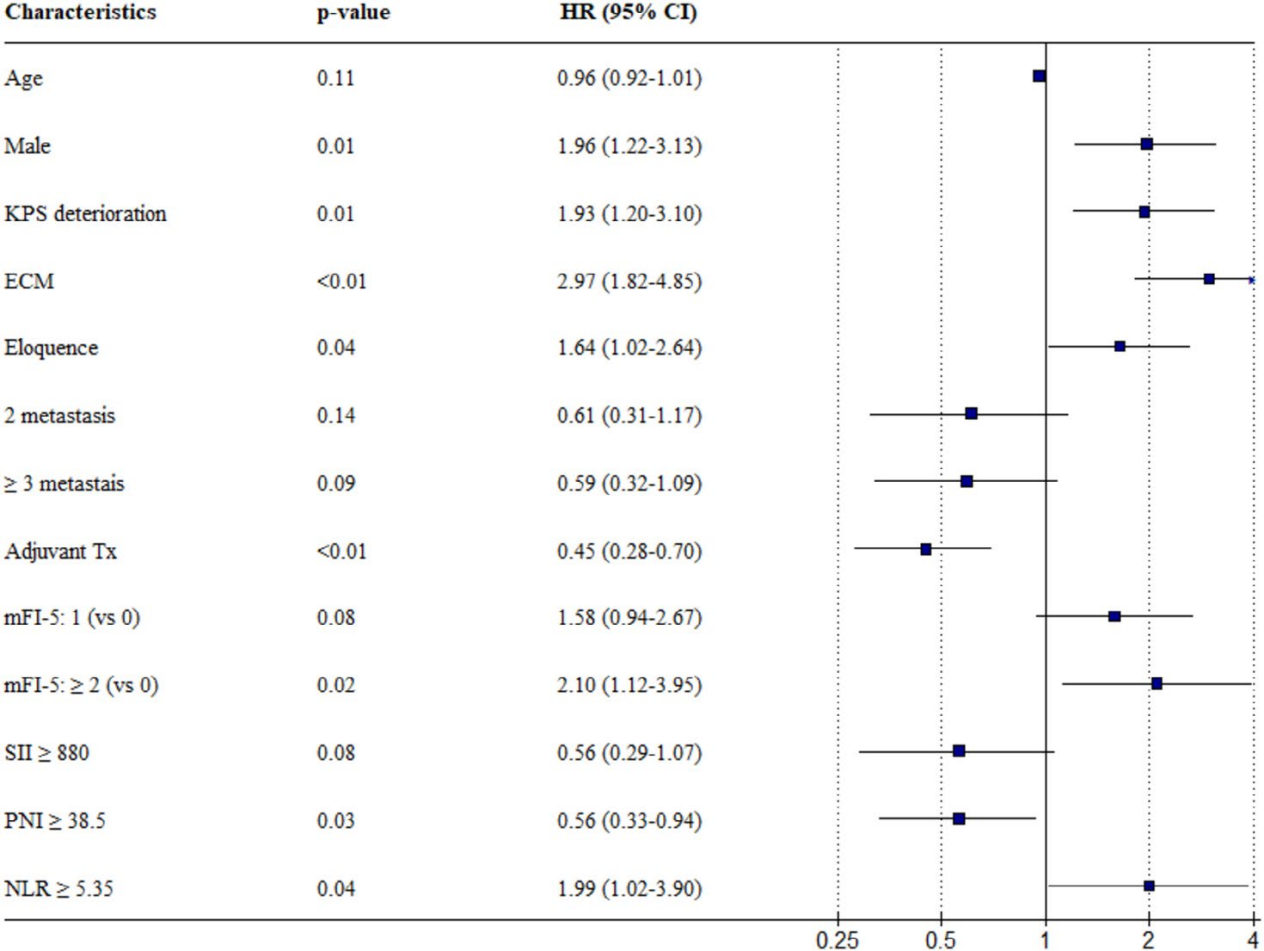
Multivariate Cox regression analysis of prognostic factors.

After variable selection using the AIC method, the variables included were controlled age, male sex, ECM, metastasis in the eloquent area, KPS deterioration, number of BM, post‐BM systemic treatment status, mFI‐5, SII, PNI, and NLR. In the multivariate analysis, male sex (HR: 1.96, 95% CI: 1.22–3.13), ECM (HR: 2.97, 95% CI: 1.82–4.85), BM in eloquent locations (HR: 1.64, 95% CI: 1.02–2.64), KPS deterioration (HR: 1.93, 95% CI: 1.20–3.10), and mFI‐5 equal to or greater than 2 (HR: 2.10, 95% CI: 1.12–3.95) were associated with poor overall survival. Conversely, receiving systemic treatment after the diagnosis of BM showed a significant overall survival benefit (HR: 0.45, 95% CI: 0.28–0.70). Elevated SII (HR: 1.99, 95% CI: 1.02–3.90) was significantly associated with poor survival, while elevated PNI (HR: 0.56, 95% CI: 0.33–0.94) indicated better survival. Detailed information is provided in Table 2 and Figure 3.

### 3.5 Subgroup Analysis

**FIGURE 4:**
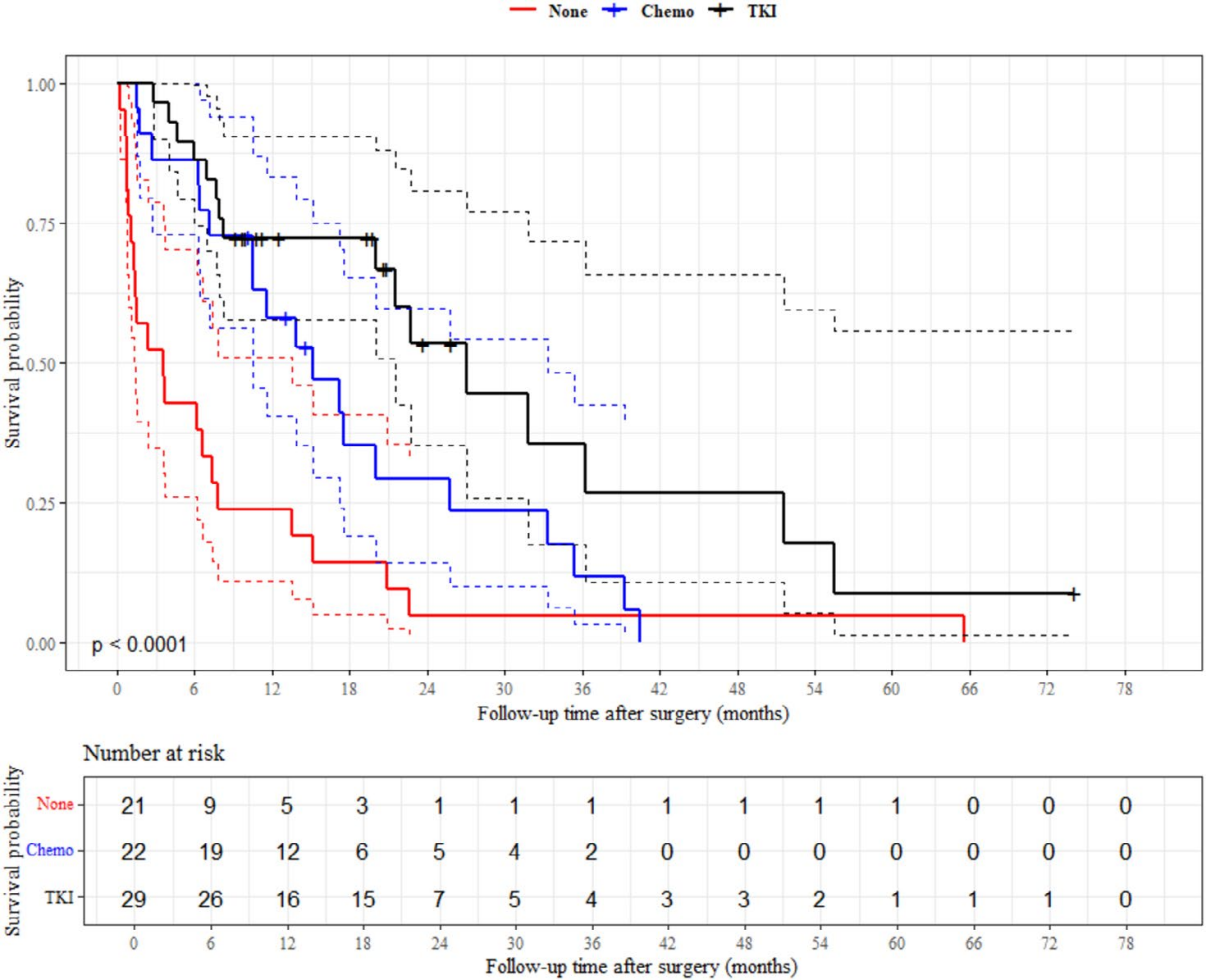
Kaplan–Meier survival curve of non‐small cell lung cancer patients according to their systemic therapy after surgery.

We conducted a subgroup analysis for NSCLC. Male sex (HR: 2.83, 95% CI: 1.28–6.26), presence of ECM (HR: 2.81, 95% CI: 1.35–5.85), mFI‐5 being 1 (HR: 2.34, 95% CI: 1.09–5.00), and mFI‐5 being equal to or greater than 2 (HR: 3.30, 95% CI: 1.42–7.69) were associated with poor overall survival. Conversely, the administration of systemic treatment after the diagnosis of BM showed an overall survival benefit (HR: 0.46, 95% CI: 0.24–0.88), as did the administration of targeted therapy (TKI) (HR: 0.25, 95% CI: 0.13–0.48) and chemotherapy (HR: 0.47, 95% CI: 0.25–0.90) (Figure 4). Elevated PNI (HR: 0.47, 95% CI: 0.23–0.99) was also associated with better overall survival. Due to the reduction in sample size, several variables became insignificant, but the direction of the hazard ratio remained similar to the total analysis. Detailed information is provided in Table S1.

## 4 Discussion

Our findings demonstrated that male sex, ECM, BM in eloquent locations, postoperative deterioration in KPS, increased mFI‐5 score, and elevated SII were independent predictors of shorter overall survival. Moreover, patients who received postoperative systemic treatment had longer survival. Patients with higher preoperative PNI had better survival. These findings underscore the importance of considering various prognostic factors and the potential effect of postoperative systemic treatment in elderly patients with BM.

Sexual dimorphisms have garnered increasing attention in cancer research. Jung et al. found no significant association between sex and survival outcomes in patients with CRC and BM [25]. Similarly, in a study by Sperduto et al., which included patients with BM from RCC, sex did not affect survival [26]. However, our study revealed that female patients may have higher survival rates than male patients. Since our study focused on the elderly population, with the majority having NSCLC, the better survival among females was consistent, even in the NSCLC subgroup. This is consistent with a study by Barquín et al., where they observed that females with stage IV NSCLC tend to have longer survival, with a 33% increase in survival time compared to males [27]. However, in more economically developed regions, women generally have a higher life expectancy than men. Therefore, our significant finding may simply reflect the natural difference in life expectancy between men and women [28].

In our previous meta‐analysis, we identified that the presence of ECM was a significant predictor of poor survival in patients with BM from CRC [11]. Yen et al. reported that patients with BM from NSCLC who underwent surgical resection had shorter survival if they exhibited ECM preoperatively [29]. Consistent with these findings, our study focusing on elderly patients revealed that those without ECM who underwent BM surgery had longer survival times than those with ECM. The presence of ECM likely indicates a more aggressive and widespread disease, which can compromise the overall effectiveness of localized treatments such as surgery for BM. Furthermore, our results demonstrated that patients who received postoperative systemic therapy exhibited better survival outcomes. This observation may be attributed to improved control of primary cancer and ECM through systemic therapies, which target both intracranial and extracranial diseases.

Functional outcomes were used to assess the overall impact of the surgical intervention on the patient's daily life and well‐being. In a recent study conducted by Wolfert et al., the use of radio‐oncological scores containing functional status components to stratify patients undergoing surgery for BM was investigated, which revealed that these preoperative scores, such as the score index of radiosurgery, graded prognostic assessment, and recursive partitioning analysis (RPA), have the potential to predict survival outcomes following surgery [30]. Our study included patients older than 65 years with brain metastasis, classified as RPA 2 or above. Patients with preoperative KPS < 70 were considered RPA 3, and our analysis confirmed that RPA 3 had a worse survival outcome. However, we would like to emphasize that even if preoperatively they were not functionally dependent, postoperative deterioration in performance status was significantly associated with worse survival. Postoperative neurological deterioration can arise from various factors, including intraoperative surgical manipulation or direct damage to the brain tissue, severe peritumoral brain edema, arterial ischemia, venous infarction, perioperative seizures, and other comorbidities, with an incidence ranging from 7% to 20% [31]. Our results revealed a significant association between postoperative KPS score deterioration and poor survival. Evidence has shown that performance deterioration following neurosurgical procedures is associated with higher mortality rates, functional dependency, and poorer outcomes in terms of disability, cognition, depression, and quality of life, particularly among elderly patients [32]. Therefore, neurosurgeons should take appropriate measures to mitigate the likelihood of neurological deterioration and optimize patient outcomes.

Mitsuya et al. analyzed patients with BM from several primary sites and showed that NLR > 5 is a predictor of worse survival [33], which is attributed to the stimulation of tumor angiogenesis and the proliferation of neoplasms caused by increased neutrophils. The PLR is an informative marker of shifts in platelet and lymphocyte counts due to acute inflammatory and prothrombotic states in several types of cancers [34]. However, in our study, these two biomarkers were not significantly associated with survival. The SII is another biomarker based on lymphocyte, neutrophil, and platelet counts. Li et al. reported that SII is an independent prognostic factor in BM from lung cancer [35]. In our study, a higher SII was associated with a poor survival outcome in the multivariate analysis. The elevation of the SII may potentially explain our results, as it could indicate more severe immune suppression, inflammatory responses, and tumor‐related angiogenesis [36]. The PNI serves as an indicator of the immunonutritional status, with emerging studies suggesting its potential as a protective biomarker of cancer prognosis [37, 38]. In our study, results align with the previous literature, indicating that patients with a higher PNI have a better nutritional status, which results in a stronger immune response.

The mFI‐5 was used to determine the frailty of the patients with allocated 1 point for each of the following criteria: congestive heart failure, chronic obstructive pulmonary disease or recent pneumonia, hypertension requiring medication, diabetes mellitus, and non‐independent functional status [39]. Heimann et al. grouped elderly patients undergoing BM surgery into least‐frail, moderately frail, and frailest groups, in which the frailest group exhibited significantly worse survival outcomes [12]. Similar to the aforementioned study, our multivariate analysis showed that an mFI‐5 score being equal to or greater than 2 had a worse prognostic effect on survival after surgery.

### 4.1 Clinical Implications

In addition to determining whether the tumor is located in an eloquent area, it is important to assess the presence of ECM and comorbidity using the mFI‐5, as these factors can predict prognosis. ECM indicates a more aggressive disease course and higher tumor burden, while associated comorbidities can complicate treatment and recovery. Evaluating whether patients are suitable for postoperative systemic treatment is crucial, as systemic therapies can target residual microscopic disease and improve overall survival. Preoperative SII and PNI should be assessed, as inflammation can promote tumor growth and metastasis through various pathways, including the release of cytokines and growth factors that enhance tumor cell proliferation, and better nutritional status is associated with improved survival outcomes. Monitoring the performance status both preoperatively and postoperatively is essential, as deterioration in functional status is associated with shorter survival. Functional decline can be a result of the direct effects of the tumor in the brain and the impact of surgery and other treatments on the patient's overall health. Patients experiencing postoperative functional decline may require tailored rehabilitation programs and supportive care to improve functional outcomes.

### 4.2 Limitations

In addition to the inherent limitations of the retrospective study design, several shortcomings should be addressed. First, the study's retrospective nature introduces potential selection bias, as the inclusion of patients who underwent surgery for BM may not adequately represent the general population of elderly patients with BM, because their conditions may inherently differ from those who did not undergo surgery. Second, the sample size is rather small, and the majority of cancer types were NSCLC and CRC, with a small number of patients with other cancer types, compromising the generalizability of the results to elderly patients with BM with various primary cancers. Third, in patients with surgically treated BM, perioperative seizures were another important factor associated with postoperative outcomes [40]. However, the administration of preventive antiepileptic drugs was determined by the individual surgeons' experience rather than a standardized protocol in our department. This introduces potential bias in the actual incidence of perioperative seizures, making it challenging to analyze the relationship between perioperative seizures and survival outcomes. Fourth, the heterogeneity of the patient population, including differences in primary cancer types, disease stages, and treatment histories, may have influenced the study outcomes and limited the ability to draw definitive conclusions. Fifth, since we investigated the association between functional outcome and survival following BM surgery, it would have been more precise to determine whether the cause of mortality was associated with postoperative functional deterioration or progression of malignancy. With limited medical records, we were unable to differentiate the causes of mortality between the two factors in our study. Finally, we included patients from 2010 to 2023, and survival outcomes may have been influenced by improvements in diagnostic and therapeutic modalities during this period, which are potential confounders that could not be accounted for.

## 5 Conclusions

Clinicians should adopt a personalized approach when selecting treatment options for elderly patients with BM, considering BM locations, the presence of ECM, comorbidities, and suitability for postoperative systemic treatment. Evaluating preoperative nutritional and inflammatory status and monitoring performance status pre‐ and postoperatively are needed, as these factors may affect prognosis.

## Conflicts of Interest

The authors declare no conflicts of interest.

## Supporting information


**Table S1:** Subgroup analysis for NSCLC.

## Data Availability

The data that support the findings of this study are available from the corresponding author upon reasonable request.
