# Correction to “Prognostic Factors Impacting Surgical Resection Outcomes in Elderly Patients With Brain Metastasis”

**DOI:** 10.1002/kjm2.70246

**Published:** 2026-06-07

Y. Chang , H.‐J. Hsu , C.‐E. Wong , et al., “Prognostic Factors Impacting Surgical Resection Outcomes in Elderly Patients With Brain Metastasis,” The Kaohsiung Journal of Medical Sciences 42, no. 2 (2026): e70099, 10.1002/kjm2.70099.40855791 PMC12884738

Upon review and verification of the patient enrollment records, we identified an error in the description of the study period in the Methods section.

The study period was incorrectly stated as “between January 2010 and December 2023.”

The correct study period should be “between January 2011 and July 2022.”

This correction is limited to the description of the study period and does not affect the study data, statistical analyses, results, or conclusions of the article.

We apologize for this error.

