# Supplementary material for: Prognostic Factors Impacting Surgical Resection Outcomes in Elderly Patients With Brain Metastasis
Source: Kaohsiung J Med Sci. 2025 Aug 26;42(2):e70099. doi: 10.1002/kjm2.70099 (PMC12884738; doi:10.1002/kjm2.70099)
Supplement: Supplementary file 1 — Table S1: Subgroup analysis for NSCLC. [file KJM2-42-e70099-s001.docx]

| **Supplemental Table 1. Subgroup analysis for NSCLC** | | | | | | |
| --- | --- | --- | --- | --- | --- | --- |
| Characteristics |  | Univariate Cox regression |  |  | Multivariate Cox regression |  |
|  | HR | 95% CI | *p*-Value | HR | 95% CI | *p*-Value |
| Age | 1.03 | 0.97-1.09 | 0.354 | 0.98 | 0.91-1.05 | 0.548 |
| Male | 1.97 | 1.11-3.49 | 0.021* | 2.83 | 1.28-6.26 | 0.01* |
| RPA:3 | 1.70 | 0.94-3.10 | 0.081 |  |  |  |
| Controlled primary tumor status | 0.87 | 0.50-1.52 | 0.628 |  |  |  |
| ECM | 1.50 | 0.85-2.62 | 0.166 | 2.81 | 1.35-5.85 | 0.006* |
| Eloquence | 1.74 | 1.00-3.01 | 0.050* | 1.76 | 0.89-3.46 | 0.102 |
| KPS deteriorate | 1.87 | 1.09-3.23 | 0.024* | 1.42 | 0.72-2.79 | 0.314 |
| BM bleeding | 1.23 | 0.69-2.17 | 0.481 |  |  |  |
| Number of BM |  |  |  |  |  |  |
| 1 | Ref |  |  |  |  |  |
| 2 | 1.33 | 0.67-2.65 | 0.419 | 0.63 | 0.26-1.57 | 0.325 |
| ≥ 3 | 0.79 | 0.41-1.55 | 0.501 | 0.64 | 0.29-1.42 | 0.272 |
| Synchronous BM | 1.24 | 0.73-2.12 | 0.43 |  |  |  |
| Infratentorial involvement | 0.96 | 0.40-2.27 | 0.918 |  |  |  |
| Tumor size (cm) | 1.00 | 0.98-1.02 | 0.808 |  |  |  |
| Edema size (cm) | 0.99 | 0.98-1.01 | 0.357 |  |  |  |
| Pre BM systemic tx | 0.75 | 0.44-1.26 | 0.275 |  |  |  |
| Post BM systemic tx | 0.39 | 0.23-0.67 | < 0.001* | 0.46 | 0.24-0.88 | 0.019* |
| Chemotherapy (vs. no adjuvant treatment) | 0.47 | 0.25-0.90 | 0.022* |  |  |  |
| TKI (vs. no adjuvant treatment) | 0.25 | 0.13-0.48 | <0.001* |  |  |  |
| Functional dependence | 1.79 | 1.00-3.22 | 0.051 |  |  |  |
| mFI-5 |  |  |  |  |  |  |
| 0 | Ref |  |  |  |  |  |
| 1 | 2.07 | 1.06-4.00 | 0.032* | 2.34 | 1.09-5.00 | 0.029* |
| ≥ 2 | 2.05 | 1.07-3.93 | 0.031* | 3.3 | 1.42-7.69 | 0.006* |
| SII ≥ 880 | 1.41 | 0.81-2.45 | 0.222 | 1.57 | 0.70-3.53 | 0.275 |
| PNI ≥ 38.5 | 0.52 | 0.29-0.94 | 0.030* | 0.47 | 0.23-0.99 | 0.047* |
| PLR ≥ 301.5 | 1.29 | 0.70-2.38 | 0.419 |  |  |  |
| NLR ≥ 5.35 | 1.16 | 0.67-2.01 | 0.607 | 0.70 | 0.31-1.60 | 0.402 |
| EGFR mutation | 0.75 | 0.44-1.29 | 0.303 |  |  |  |
| BM, brain metastasis, CI, confidence interval, ECM, extracranial, HR, hazard ratio, KPS, Karnofsky performance status, TKI, tyrosine kinase inhibitor, mFI-5, modified 5-item frailty index, NLR, neutrophil-to-lymphocyte ratio, PLR, platelet-to-lymphocyte, PNI, prognostic nutrition index, RPA, recursive partitioning analysis, SII, systemic inflammation index, *statistically significant | | | | | | |
